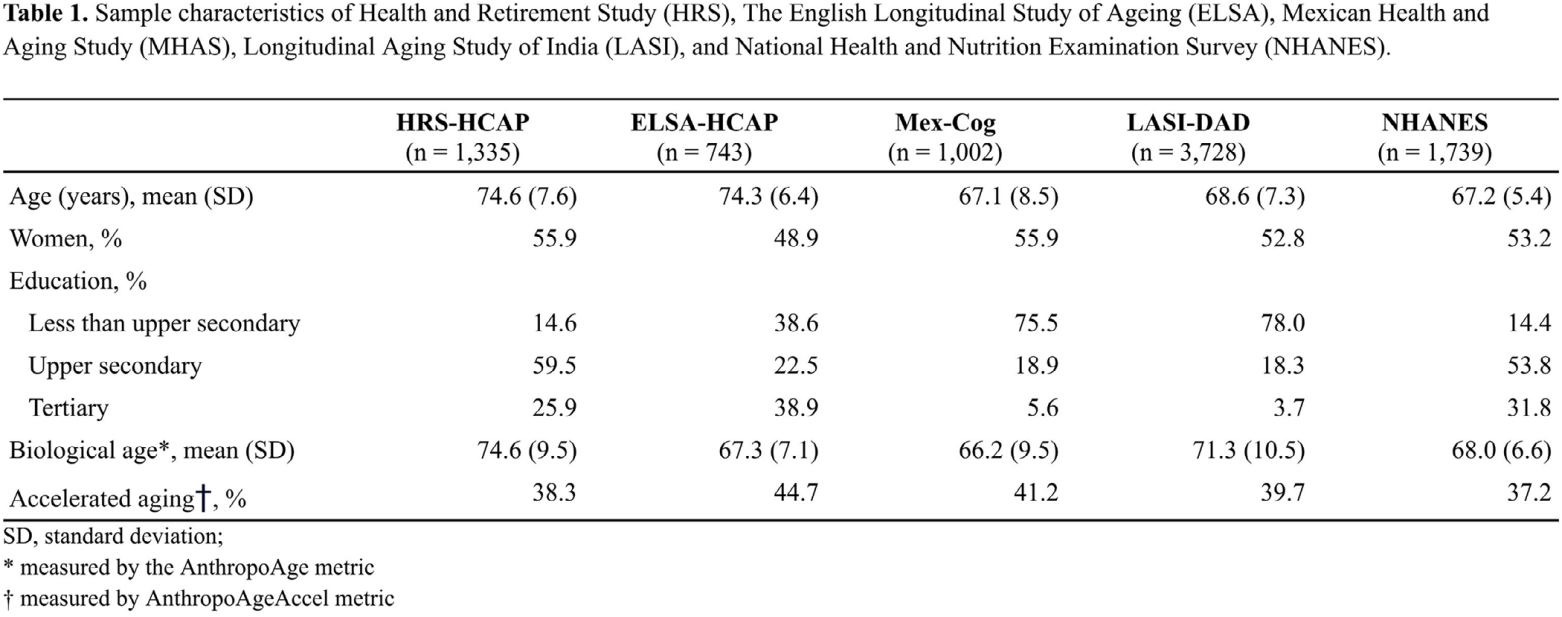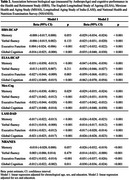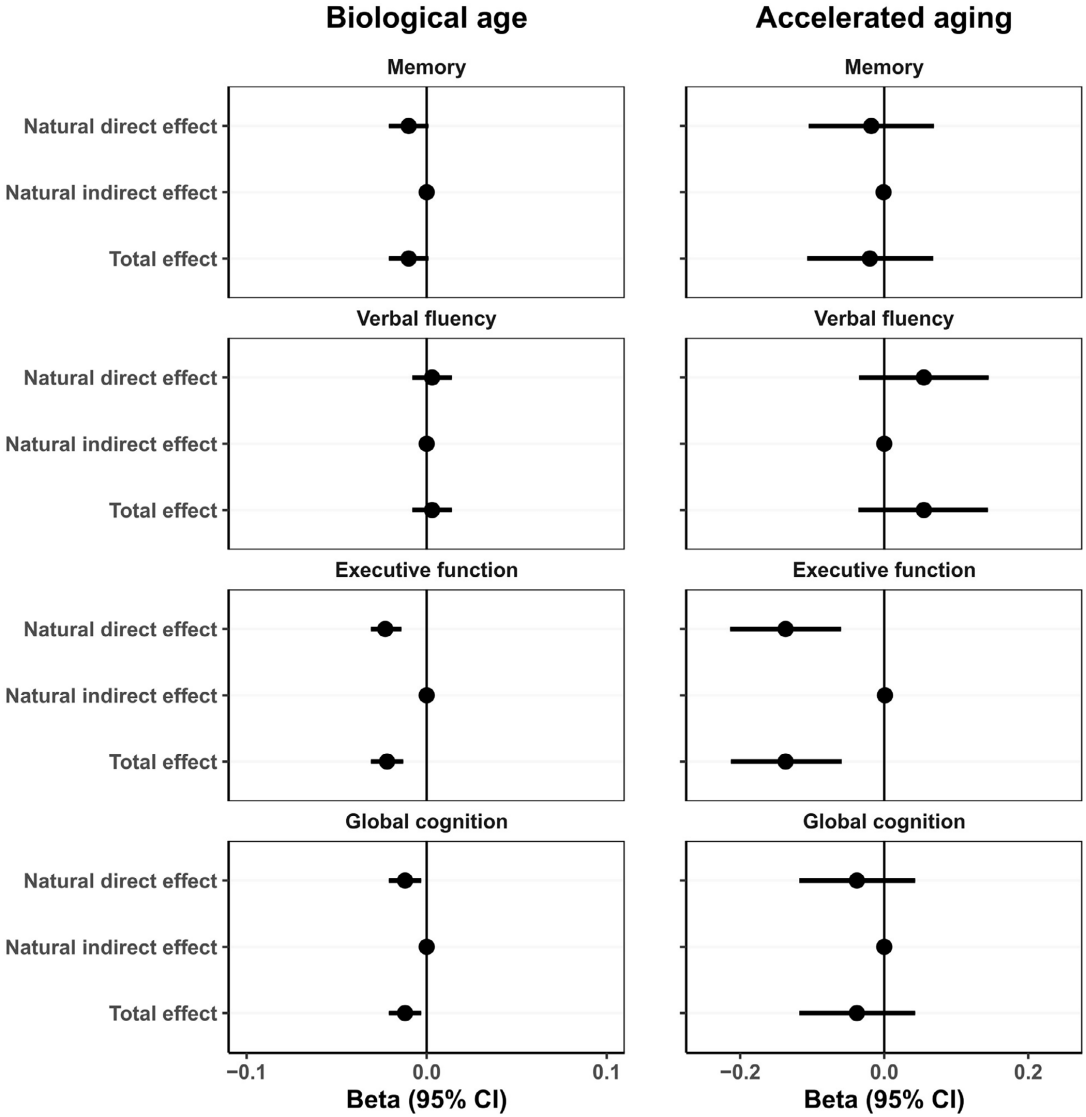# Accelerated biological aging is associated with cognitive performance among older adults in Mexico, India, United States and England: A cross‐national retrospective analysis

**DOI:** 10.1002/alz70860_099116

**Published:** 2025-12-23

**Authors:** Ingryd Mayara Nascimento Martins‐Pais, Carlos Fermín‐Martínez, Omar Bello‐Chavolla, Paulo Henrique Lazzaris Coelho, Naomi Vidal‐Ferreira, Claudia Kimie Suemoto, Natalia Gomes Goncalves

**Affiliations:** ^1^ Universidade de Sao Paulo, Sao Paulo, SP, Brazil; ^2^ Research Division, Instituto Nacional de Geriatría, Mexico City, EM, Mexico; ^3^ Instituto Nacional de Geriatría, Ciudad de México, EM, Mexico; ^4^ University of São Paulo Medical School, São Paulo, SP, Brazil; ^5^ Adventist College of Amazonia, Benevides, Pará, Brazil; ^6^ Division of Geriatrics, University of São Paulo Medical School, São Paulo, Brazil

## Abstract

**Background:**

Age is a major risk factor for cognitive decline; however, chronological age alone cannot capture the complex biological, genetic, and environmental changes involved in aging. A novel biological age metric based on anthropometric data, AnthropoAge, was recently developed and was shown to be a better predictor of mortality than chronological age. However, its relationship with cognitive performance remains unexplored. Furthermore, the anti‐aging protein Klotho may mediate aging effects through metabolic and anti‐inflammatory pathways. This study aimed to investigate the association between AnthropoAge and cognitive performance in global samples of older adults and whether Klotho mediated this association.

**Method:**

The sample included data from participants aged 60 years or older from the National Health and Nutrition Examination Survey (NHANES), and the Harmonized Cognitive Assessment Protocol (HCAP) from the Health and Retirement Study (HRS‐HCAP), English Longitudinal Study on Ageing (ELSA‐HCAP), Longitudinal Aging Study in India (LASI‐DAD), and Mexican Health and Aging Study (Mex‐Cog). The simplified version of AnthropoAge, which includes chronological age in years, body mass index, and waist‐to‐height ratio, was used. Cognitive performance was assessed through memory, executive function, and verbal fluency, with a global composite z‐score derived from the tests. Linear regression models were used to examine the associations between AnthropoAge and cognition, and mediation analysis was conducted to test whether this association was mediated by Klotho.

**Result:**

Data from 1,335 HRS participants (55.9% women), 743 ELSA participants (48.9% women), 1,002 MHAS participants (55.9% women), 3,728 LASI participants (52.8% women), and 1,739 NHANES participants (53.2% women) were analyzed (Table 1). The average biological age of participants was 74.6, 67.3, 66.2, 71.3, and 68.0 years, respectively (Table 1). In these cohorts, higher biological age measured by AnthropoAge was associated with poorer cognitive performance across all countries evaluated (Table 2). However, these associations were not mediated by Klotho (Figure 3).

**Conclusion:**

Higher biological age measured by AnthropoAge was associated with poorer cognitive performance, with the specific cognitive domains impacted differing by country. Klotho did not mediate this association. Implementing this metric at the population level could help identify individuals at higher risk of poorer cognitive performance, facilitating targeted interventions.